# Intraoperative Management of the Recurrent Laryngeal Nerve Transected or Invaded by Thyroid Cancer

**DOI:** 10.3389/fendo.2022.884866

**Published:** 2022-06-09

**Authors:** Hiroo Masuoka, Akira Miyauchi

**Affiliations:** Department of Surgery, Kuma Hospital Center for Excellence in Thyroid Care, Kobe, Japan

**Keywords:** Recurrent Laryngeal Nerve, reconstruction, Intraoperative neural monitoring (IONM), laryngeal approach, partial layer resection

## Abstract

Thyroid cancer often invades the recurrent laryngeal nerve (RLN), causing vocal cord paralysis. In such patients, the invaded portion of the RLN usually needs to be resected through curative surgery. We attempt to preserve the nerve by performing sharp dissection in such cases. During nerve dissection, an intraoperative nerve monitoring system helps identify the course of the RLN in the fibrous tissue around the tumor or even within the tumor, and also helps evaluate the nerve integrity. Because of extensive dissection, the preserved RLN may become much thinner than its original thickness. We refer to this procedure as “partial layer resection” of the RLN. In our cases, although the dissected RLNs became thinner, we found that vocal cord function recovered in most patients. If the RLN is fully involved by thyroid cancer or response of the vocal cord against electric stimulation to the RLN is lost, we resect the portion of the RLN together with the tumor and repair it using one of the reconstruction techniques. When a unilateral RLN is resected, the vocal cord on that side is paralyzed. Symptoms include hoarseness, mis-swallowing, and short phonation. RLN reconstruction using one of the reconstruction techniques leads to the recovery of phonatory and swallowing function, although the normal motion of the vocal cord on the side of the anastomosis is not restored. We used direct anastomosis, free nerve grafting, ansa cervicalis-RLN anastomosis, and vagus-RLN anastomosis to reconstruct the RLN. Thyroid cancer often invades the RLN near the Berry’s ligament. In such patients, surgeons might assume that reconstruction of the RLN may not be possible because the peripheral stump of the RLN cannot be observed. However, if we divide the inferior pharyngeal constrictor muscles along the lateral edge of the thyroid cartilage, the peripheral RLN can be identified, and nerve reconstruction can be performed. We refer to this procedure as “laryngeal approach”.In summary, of the patients with thyroid cancer who required resection of the RLN, RLN reconstruction led to the recovery of phonatory function. We suggest that all thyroid surgeons familiarize themselves with these reconstruction techniques.

## Introduction

Thyroid cancer often invades the recurrent laryngeal nerve (RLN), causing vocal cord paralysis (VCP). In such patients, the invaded portion of the RLN usually needs to be resected through curative surgery. Even in patients with functional vocal cords preoperatively, RLN may be involved when performing thyroid surgery for thyroid cancer. We attempt to preserve the nerve by performing sharp dissection in such cases. Nishida et al. reported that preservation of the RLN invaded by differentiated thyroid cancer with sharp dissection rarely causes local recurrence (1). Our results are in accordance with those of their report.

In recent years, we have routinely used an intraoperative neural monitoring system to evaluate the integrity of the inferior laryngeal nerve (RLN) and external branches of the superior laryngeal nerve during thyroid surgery (2). This system helps assess the electrophysiological response of the RLN prior to dissection. If the response is normal, we try preserving the RLN by shaving off the tumor (3). During nerve dissection, an intraoperative nerve monitoring system helps identify the course of the RLN in the fibrous tissue around the tumor or even within the tumor and also helps evaluate the nerve integrity. If the response is weakened or lost, we resect the portion of the RLN together with the tumor and repair it using one of the reconstruction techniques described below.

In the personal case series of one of the authors (Miyauchi A.), among 721 primary thyroid cancer cases treated between 1998 to 2008, 4.3% of the patients presented with VCP preoperatively, and even in patients with functioning vocal cords preoperatively, 2.2% required resection of the RLN because of cancer invasion. Therefore, in total, 6.4% of the patients required resection of the RLN (**Table 1**). If a unilateral RLN is severed or fully injured, the vocal cord on that side is paralyzed. It remains fixed in a paramedian position, becomes atrophied, and loses its tension during phonation. Symptoms include hoarseness, mis-swallowing, and short phonation due to the waste of exhaled air during phonation. Aspiration can be a dangerous symptom, particularly among elderly patients.

**Table 1:** Number (%) of patients who required resection of the recurrent laryngeal nerve in A.M.’s personal series from 1998 to 2008 consisting of primary thyroid cancer cases.

	VCP preoperatively		
	No	Yes	Total
No. of patients	690 (95.7%)	31 (4.3%)	721 (100.0%)
Patients with RLN resection	16 (2.2%)	30 (4.2%)	46 (6.4%)

VCP, vocal cord paralysis.

We reported vocal improvement after various methods of RLN reconstruction, although normal vocal cords movements were not restored (4, 5).

Here, we describe how to manage the RLN invaded by thyroid cancer.

## Partial Layer Resection of the RLN

**Figure 1:**
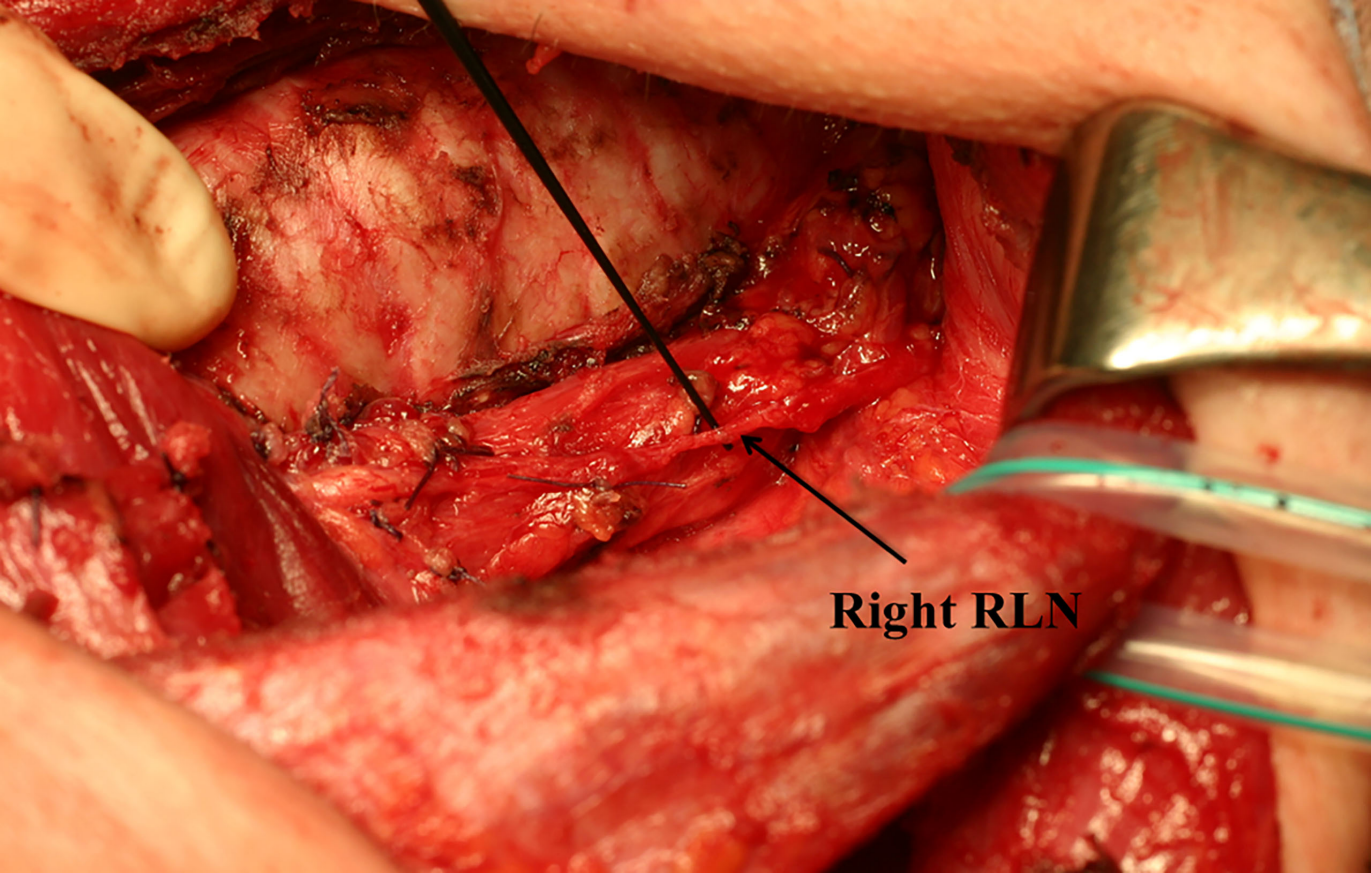
The recurrent laryngeal nerve after partial layer resection. Due to extensive resection, the recurrent laryngeal nerve became much thinner than its original thickness.

**Figure 2:**
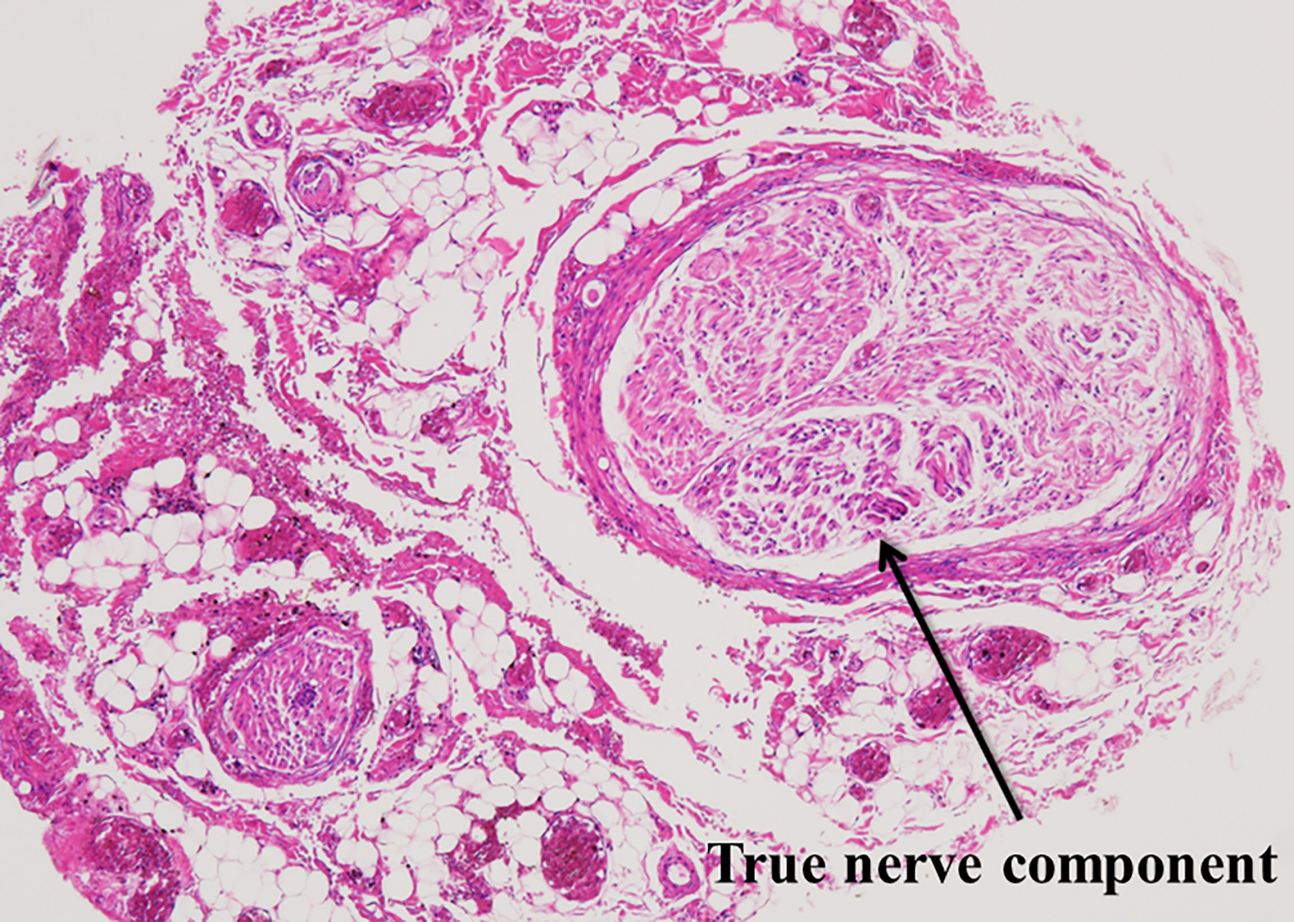
Microscopic cross-section of the normal portion of the recurrent laryngeal nerve resected due to invasion by thyroid cancer. The true nerve component is surrounded by thick peri-neural tissue.

Because of extensive dissection, the preserved RLN may become much thinner than its original thickness (**Figure 1**). We refer to this procedure as “partial layer resection” of the RLN. In our case, although the dissected RLNs became thinner, we found that vocal cord function recovered in most patients. We reviewed our medical records and found that 18 patients underwent partial layer resection of the RLN to remove thyroid cancer. Postoperatively, two (11%) patients had no VCP, 13 (72%) patients had temporary VCP followed by full recovery of vocal cord function, and only three (17%) patients had permanent VCP (6). These unexpected outcomes prompted us to study the anatomy of the RLN. We studied the histology of the cross-sections of the normal portions of RLNs resected due to thyroid cancer invasion. We found that the true nerve component was surrounded by thick perineural tissue (**Figure 2**). This anatomy may explain the favorable outcomes obtained after partial layer resection of RLNs (6). Even in cases where the preserved RLN becomes thinner than its original thickness following extensive shaving, we concluded that it is worth preserving the nerve if it was functioning well preoperatively.

## Reconstruction of the Transected or Resected RLN

### Methods of Reconstruction of the RLN

**Figure 3:**
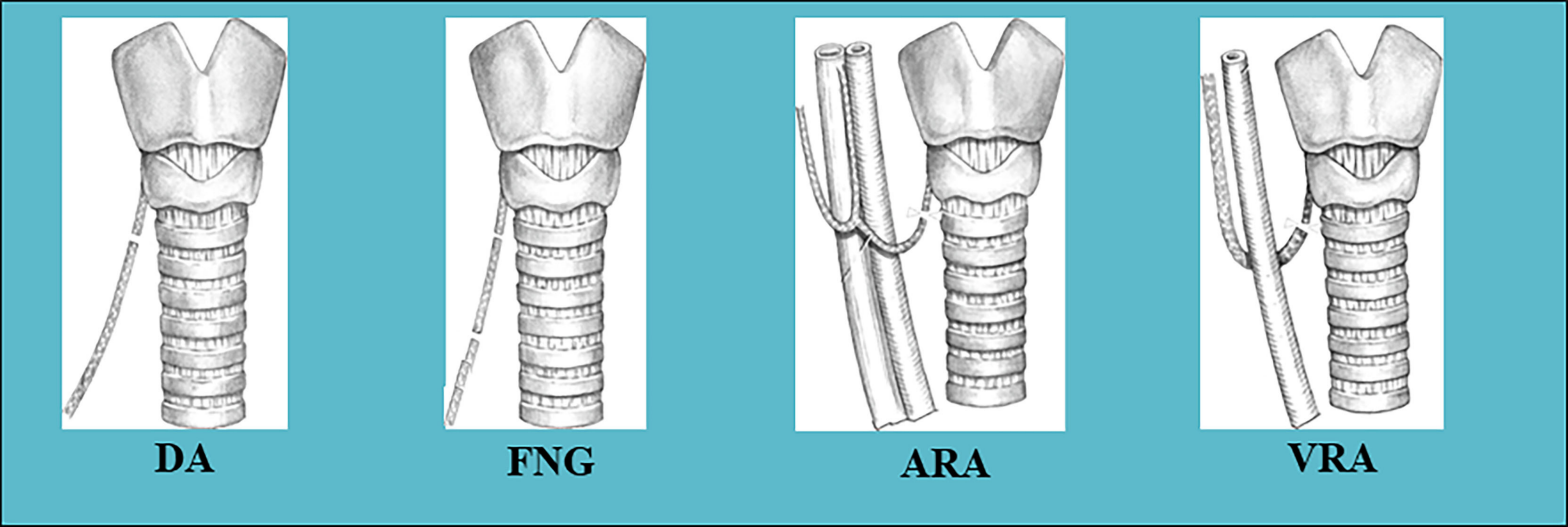
Methods of reconstruction of the RLN used in this series: DA, direct anastomosis; FNG, free nerve grafting; ARA, ansa cervicalis-RLN anastomosis; VRA, vagus-RLN anastomosis.

In 2009, Miyauchi A., one of the co-authors in this paper, reported improvement in phonation following RLN reconstruction performed during thyroid surgery in 88 patients with thyroid cancer invading the nerve in his personal series from 1984 to 2007 at Kuma Hospital or Kagawa Medical University Hospital (7). The patients included 72 women and 16 men aged 18−78 years with a mean age of 55.6 years. Of the patients, 51 (58%) underwent preoperative VCP. Seven patients underwent a second surgery for recurrent disease, while the others were primary cases. We used direct anastomosis (DA), free nerve grafting (FNG), ansa cervicalis-RLN anastomosis (ARA), and vagus-RLN anastomosis (VRA) to reconstruct the RLN (**Figure 3**). ARA was performed in most patients (n=65; 74%). DA was performed in only seven patients, FNG was performed in 14 patients, and VRA was attempted in two patients in whom both the unilateral RLN and its ipsilateral vagus nerve required resection due to cancer invasion. In 3 FNG cases and 31 ARA cases, anastomosis was performed behind the thyroid cartilage. In 8 ARA cases, anastomosis was performed using the contralateral ansa cervicalis. Surgical loupes at 2.5 × magnification were used in 51 patients. The operating microscope was used in 10 patients, and no magnifier was used in 27 patients. The thickness of the thread used for anastomosis ranged from 6-0 to 9-0, with 8-0 being the most common thickness. He generally used three stitches for end-to-end anastomosis of the nerves and used microsurgical instruments to construct the anastomosis. At Kuma Hospital, we use 8-0 monofilament thread and microsurgery instruments and encouraged the use of surgical loupes at 2.5 × magnification.

**Figure 4:**
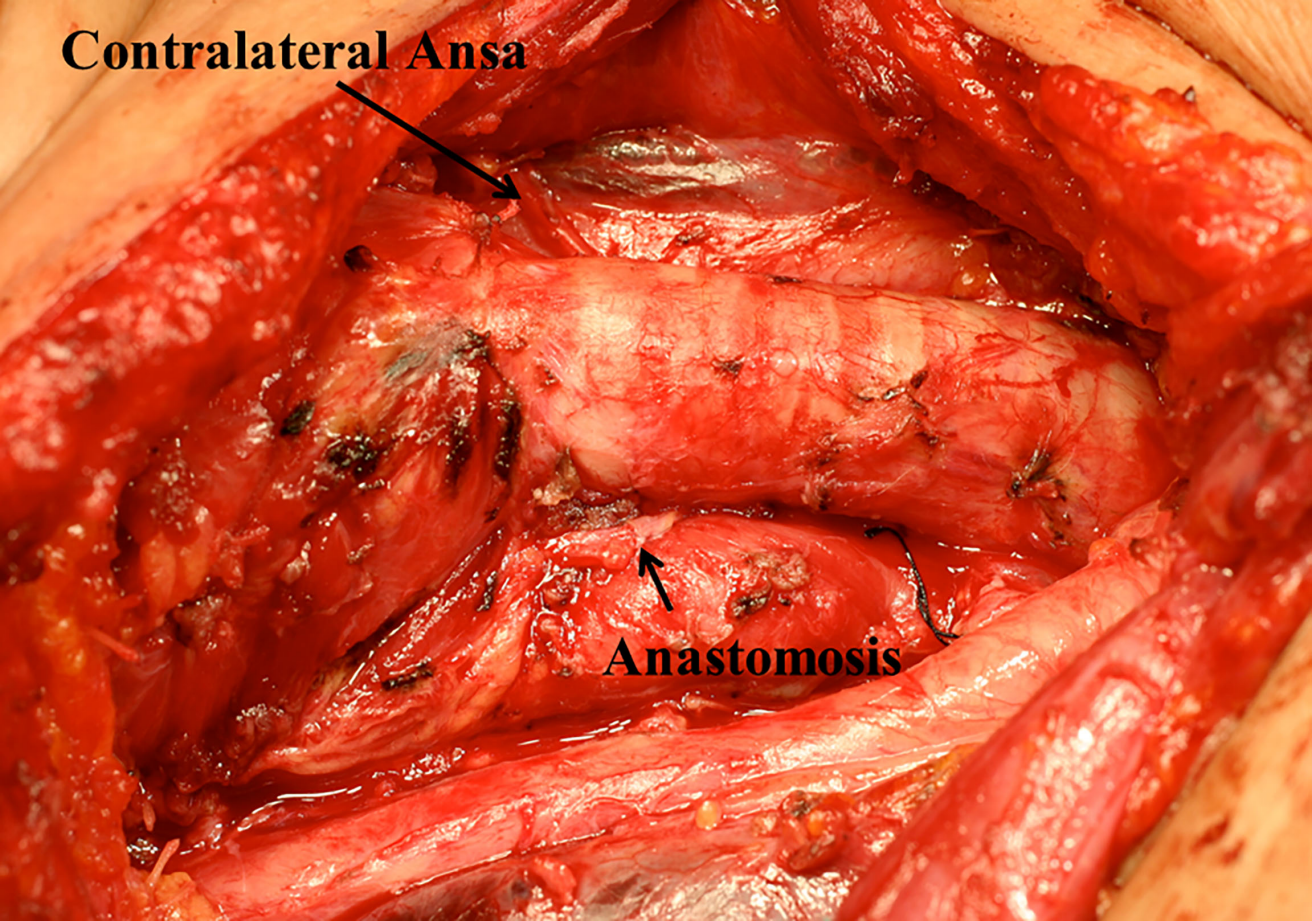
Contralateral ansa cervicalis-to-RLN anastomosis: The contralateral ansa-cervicalis was brought to the side of the defected RLN between the trachea and the esophagus to make contralateral ansa cervicalis-to-RLN anastomosis.

In patients with extensive node metastases, the ansa cervicalis on the same side may not be available. In such cases, the contralateral ansa cervicalis can be used for reconstruction. In cases, we inserted a suction tube between the trachea and the esophagus, sucked the contralateral ansa cervicalis into the tube, and then brought it to the side of the defect, where we made a contralateral ansa cervicalis-to-RLN anastomosis (**Figure 4**) (8). This route appears to be the shortest for performing the anastomosis. If the contralateral ansa cervicalis is too short, the FNG technique can be combined for safe anastomosis. However, this would require a longer time for reinnervation of the vocal cords and recovery during phonation. Furthermore, avoiding tension on anastomoses is key to this procedure.

### Evaluation of the Reconstructed RLN

Although we examined all patients using a fiberoptic laryngoscope after surgery, a laryngoscopic examination is not suitable for evaluating the recovery of vocal cord function because vocal cords do not return to normal motion after reconstruction of the RLN. Serial measurements of maximum phonation time (MPT) are the easiest and most practical method for evaluating recovery in phonation. We used the MPT one year after surgery to evaluate the outcomes. Thirty-four healthy subjects (26 women and eight men) and 27 patients (18 women and nine men) with unilateral VCP served as controls. Serial measurements of the MPT showed a sudden increase in MPT, usually around 3 months after surgery, when the patient’s voice improved. The MPTs were significantly shorter in patients with VCP than in healthy subjects. Normal MPT values were achieved in patients who underwent RLN reconstruction (**Table 2**) (7).

There were significant sex-specific differences in MPT in healthy subjects and in patients with VCP (**Table 2**). The MPT divided by the VC ratio (sec/L) indicates the vocal cord function of converting a unit volume of exhaled air to a certain length of phonation. This value is called the phonation efficiency index (PEI) (7). When PEIs were calculated, the sex-specific differences in healthy subjects and patients with VCP disappeared completely. It was noted that patients with VCP had significantly smaller PEIs than the healthy subjects. Nearly normal PEI values were achieved in patients who underwent RLN reconstruction (**Table 3**) (7) . Since PEI did not differ based on sex, this value is considered suitable for evaluating vocal cord function regardless of the patient’s sex. We analyzed factors that might be related to PEI one year after surgery, such as age at surgery, presence or absence of VCP preoperatively, method of RLN reconstruction, use of magnifier, and thread thickness. None of these factors significantly affected PEI one year after surgery. In general, insignificant data points did not add any value to analysis. However, in this case, they highlighted a very crucial idea. These data indicated that regardless of age or the presence/absence of VCP preoperatively, reconstruction of the transected RLN using any method/technique can help recover the patients’ voices to nearly normal levels.

**Table 2:** Maximum phonation time in healthy subjects, patients with vocal cord paralysis, and patients who underwent reconstruction of the recurrent laryngeal nerve (9).

		Healthy subjects	Patients with VCP	Patients with RLN reconstruction
Male	No. of patients	8	9	16
	MPT	28.6±10.8	10.1±4.3	20.9±11.7
Female	No. of patients	26	18	72
	MPT	16.7±5.1	6.3±2.9	18.8±6.6

MPT, maximum phonation time; values are mean ± SD (seconds). VCP, vocal cord paralysis; RLN, recurrent laryngeal nerve.

**Table 3:** Phonation efficiency index in healthy subjects, patients with vocal cord paralysis, and patients who underwent reconstruction of the recurrent laryngeal nerve (9).

		Healthy subjects	Patients with VCP	Patients with RLN reconstruction
Male	No. of patients	8	9	16
	PEI	6.79±2.52	3.24±1.49	5.53±2.72
Female	No. of patients	26	18	72
	PEI	6.73±2.04	3.29±1.48	7.59±2.82

PEI: phonation efficiency index; values are mean ± SD (seconds/L). VCP, vocal cord paralysis; RLN, recurrent laryngeal nerve.

### Laryngeal Approach

**Figure 5:**
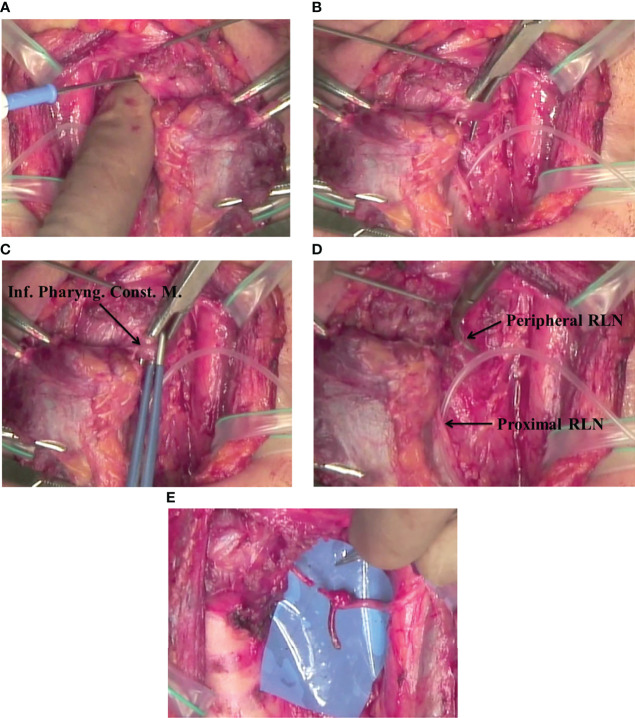
**(A)** Laryngeal approach: Marking a hole in the inferior pharyngeal constrictor muscle at the lateral edge of the thyroid cartilage using electrocautery. **(B)** Laryngeal approach: Dissection of the inferior pharyngeal constrictor muscles along the lateral edge of the thyroid cartilage. **(C)** Laryngeal approach: Cutting the inferior pharyngeal constrictor muscles along the lateral edge of the thyroid cartilage using a pair of bipolar coagulators. **(D)** Laryngeal approach: Locating the peripheral RLN behind the thyroid cartilage. **(E)** Laryngeal approach: Performing ARA with the distal portion of the RLN that was found behind the thyroid cartilage.

Thyroid cancer often invades the RLN near the Berry’s ligament. In such patients, surgeons might assume that reconstruction of the RLN may not be possible because the peripheral stump of the RLN cannot be observed. However, if we divide the inferior pharyngeal constrictor muscles along the lateral edge of the thyroid cartilage, the peripheral RLN can be identified, and nerve reconstruction can be performed (10). The anterior branch should be selected for anastomosis if possible since it usually goes to the adductor muscles. Subsequently, ARA or FNG can be performed behind the thyroid cartilage. However, finding the peripheral portion of the RLN behind the thyroid cartilage after resection of the invaded portion is challenging. We modified the surgical approach to overcome this challenge by identifying the peripheral portion of the RLN by dividing the inferior pharyngeal constrictor muscle before dissecting the nerve. We refer to this procedure as “laryngeal approach (11)”. This procedure is illustrated in F**igure 5**. Briefly, we made a small hole in the inferior pharyngeal constrictor muscle using electrocautery at the edge of the thyroid cartilage (**Figure 5A**). Next, we elevated the muscle using the jaws of a mosquito hemostat (**Figure 5B**) and cut the muscle with a pair of bipolar coagulators (**Figure 5C**). Next, we identified the peripheral portion of the RLN behind the thyroid cartilage (**Figure 5D**). This procedure makes dissection of the RLN invaded by thyroid cancer much easier because the RLN can be dissected from the central and peripheral sides. If only a portion of the RLN requires resection, the nerve can be readily reconstructed since the peripheral portion of the nerve is already identified and secured, and ansa cervicalis-to-RLN anastomosis can then be performed (**Figure 5E**) (5). For some special reasons, FNG can be used to fill the defect of the RLN, although this procedure requires two anastomoses. In addition, the divided pharyngeal muscle ends do not need to be fixed.

## Discussion

In cases where RLN is transected, surgeons can attempt its repair by direct anastomosis of the cut ends of the nerve. This is a remarkably simple idea for surgeons and was tried more than 100 years ago. The first investigators who performed this procedure reported that the movements of vocal cords were restored after anastomosis. However, other investigators later argued that this was incorrect and that recovery of vocal cord movement did not occur. As a result, anastomosis of the cut ends of the transected RLN was abandoned. However, Ezaki et al. reported that voices of seven patients recovered after direct anastomosis of the transected RLN, although their vocal cords were fixed at the median. They explained the reason behind the recovery of these patients’ voices as follows (9).

**Figure 6:**
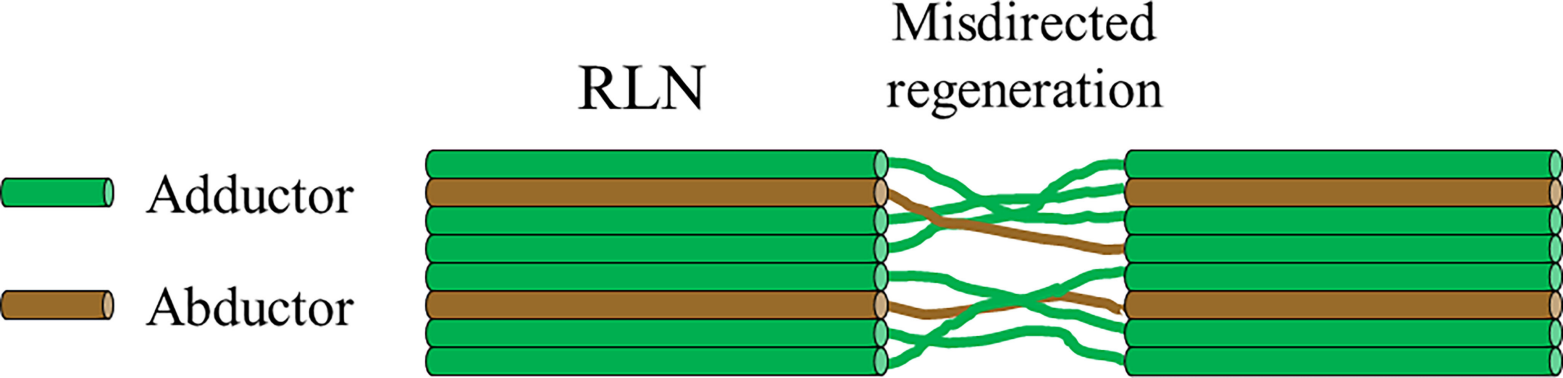
Misdirected regeneration of the recurrent laryngeal nerve. The RLN contains adductor and abductor nerve fibers without special segregation of these nerve fibers within the RLN. Following the anastomosis, the nerve fibers regenerate in a mixed fashion.

The RLN includes adductor and abductor nerve fibers without special segregation of these nerve fibers within the RLN. Following anastomosis, even if the anastomosis was performed under an operating microscope, the nerves regenerated in a mixed fashion, which is called misdirected regeneration (**Figure 6**). Therefore, the adductor and abductor muscles contract simultaneously during inspiration and phonation. In the RLN, the number of adductor nerve fibers is three times the number of abductor nerve fibers. The adductor muscles in the larynx are generally much stronger than the abductor muscles. Therefore, the normal motion of the vocal cord on the side of the anastomosis was not restored. While the cord is fixed at the median, it is not paralyzed. This condition is better expressed as synkinesis, simultaneous contraction of the adductor and abductor muscles. Following reinnervation, the vocal cord recovers from atrophy and restores tension during phonation. For good phonation, a narrow gap between the vocal cords, good tension between the vocal cords, and symmetrical volume and weight of the vocal cords are necessary. Following anastomosis, all these conditions seem to be achieved. Consequently, this improves the patient’s voice, increases phonation times, and reduced aspiration. However, paradoxical movements of the reinnervated vocal cord may occur in rare cases of extreme misdirection during the regeneration process.

DA is a simple procedure. However, this method can only be performed when the portion of the nerve defect is short or in cases of accidental transection of the RLN. Tension on the anastomosis should be avoided, and the anastomosis should be performed in an end-to-end fashion. If the defect portion is long, it can be repaired using a free nerve graft; however, this method requires two anastomoses. The graft can be harvested from the supraclavicular cutaneous nerves, transcervical nerves, auricular nerves, and ansa cervicalis, irrespective of the motor or sensory nerves. If the defect extends to the mediastinum, anastomosis at the mediastinal site may be challenging or practically impossible.

In 1990, Miyauchi et al. reported ARA as their own idea (5). However, this method was previously reported by Crumley et al. in 1986 (12). The ansa cervicalis forms a loop in front of the internal jugular vein and branches into the sternothyroid, sternohyoid, and omohyoid muscles. It is a motor nerve that is activated during phonation and respiration. The results of ARA are quite similar to those of DA of the RLN, with no recovery of vocal cord movement but recovery of phonation. Paradoxical movements of the vocal cords following ARA have never been observed. ARA requires only one anastomosis, which can be performed in an easy-to-access position of the neck near the larynx. Therefore, the time necessary for nerve regeneration and voice recovery following nerve reconstruction should also be shorter than that required for FNG. VRAs were performed in only two exceptional cases in which thyroid cancer invaded the RLN and ipsilateral vagus nerve, thus requiring resection of both nerves.

Dyspnea on inspiration can occur if bilateral RLNs are cut or injured. Depending on the degree of dyspnea, the airway may have to be opened *via* tracheostomy. Reconstruction of the RLN is not indicated for this disastrous condition because this procedure does not help restore the normal motion of the vocal cords; instead, the cords become fixated at the median position.

## Conclusion

Of the patients with thyroid cancer who needed resection of the RLN, RLN reconstruction led to the recovery of phonatory function in nearly 90% of patients. Recovery was achieved in all patients regardless of patient characteristics, presence or absence of VCP preoperatively, reconstruction method, or surgical aid. Therefore, we suggest that all thyroid surgeons familiarize themselves with these reconstruction techniques and attempt reconstructing the RLN if necessary.

## Data Availability Statement

The original contributions presented in the study are included in the article/supplementary material. Further inquiries can be directed to the corresponding author.

## Author Contributions

HM and AM designed the study. HM reviewed clinical records, and prepared the article, tables, and figures. AM revised the article. All authors contributed to the article and approved the submitted version.

## Conflict of Interest

The authors declare that the research was conducted in the absence of any commercial or financial relationships that could be construed as a potential conflict of interest.

## Publisher’s Note

All claims expressed in this article are solely those of the authors and do not necessarily represent those of their affiliated organizations, or those of the publisher, the editors and the reviewers. Any product that may be evaluated in this article, or claim that may be made by its manufacturer, is not guaranteed or endorsed by the publisher.

## References

[1] Nishida T Nakao K Hamaji M Kamiike W Kurozumi K Matsuda H. Preservation of Recurrent Laryngeal Nerve Invaded by Differentiated Thyroid Cancer. Ann Surg (1997) 226:85–91. doi: 10.1097/00000658-199707000-00012 9242342 PMC1190911

[2] Randolph GW Dralle H Abdullah H Barczynski M Bellantone R Brauckhoff M. Electrophysiologic Recurrent Laryngeal Nerve Monitoring During Thyroid and Parathyroid Surgery: International Standards Guideline Statement. Laryngoscope (2011) 121(Supplement 1):S1–16. doi: 10.1002/lary.21119 21181860

[3] Wu CW Dionigi G Barczynski M Chiang FY Dralle H Schneider R. International Neuromonitoring Study Group Guidelines 2018: Part II: Optimal Recurrent Laryngeal Nerve Management for Invasive Thyroid Cancer-Incorporation of Surgical, Laryngeal, and Neural Electrophysiologic Data. Laryngoscope (2018) 128(Supplement 3):S18–27. doi: 10.1002/lary.27360 30291765

[4] Miyauchi A Ishikawa H Matsusaka K Maeda M Matsuzuka F Hirai K. Treatment of Recurrent Laryngeal Nerve Paralysis by Several Types of Nerve Suture. J Jap Surg Soc (1993) 94:550–5. 8341239

[5] Miyauchi A Matsusaka K Kihara M Matsuzuka F Hirai K Yokozawa T. The Role of Ansa-to-Recurrent-Laryngeal Nerve Anastomosis in Operations for Thyroid Cancer. Eur J Surg (1998) 164:927–33. doi: 10.1080/110241598750005093 10029388

[6] Kihara M Miyauchi A Yabuta T Higashiyama T Fukushima M Ito Y. Outcome of Vocal Cord Function After Partial Layer Resection of the Recurrent Laryngeal Nerve in Patients With Invasive Papillary Thyroid Cancer. Surgery (2014) 155:184–9. doi: 10.1016/j.surg.2013.06.052 24646959

[7] Miyauchi A Inoue H Tomoda C Fukushima M Kihara M Higashiyama T. Improvement in Phonation After Reconstruction of the Recurrent Laryngeal Nerve in Patients With Thyroid Cancer Invading the Nerve. Surgery (2009) 146:1056–62. doi: 10.1016/j.surg.2009.09.018 19958932

[8] Miyauchi A Yokozawa T Kobayashi K Hirai K Matsuzuka F Kuma K. Opposite Ansa Cervicalis to Recurrent Laryngeal Nerve Anastomosis to Restore Phonation in Patients With Advanced Thyroid Cancer. Eur J Surg (2001) 167:540–1. doi: 10.1080/110241501316914939 11560391

[9] Ezaki H Ushio H Harada Y Takeichi N. Recurrent Laryngeal Nerve Anastomosis Following Thyroid Surgery. World J Surg (1982) 6:342–6. doi: 10.1007/BF01653553 7113239

[10] Miyauchi A. Surgical Techniques in Reconstruction of Injured RLN for Voice Rehabilitation. Tokyo: Intermerc Co. (2001).

[11] Miyauchi A Masuoka H Tomoda C Takamura Y Ito Y Kobayashi K. Laryngeal Approach to the Recurrent Laryngeal Nerve Involved by Thyroid Cancer at the Ligament of Berry. Surgery (2012) 152:57–60. doi: 10.1016/j.surg.2011.12.033 22386712

[12] Crumley RL Izdebski K. Vocal Quality Following Laryngeal Reinnervation by Ansa Hypoglossi Transfer. Laryngoscope (1986) 96:611–6. doi: 10.1288/00005537-198606000-00004 3713403

